# The Forward Effect of Testing: Behavioral Evidence for the Reset-of-Encoding Hypothesis Using Serial Position Analysis

**DOI:** 10.3389/fpsyg.2018.01197

**Published:** 2018-07-11

**Authors:** Bernhard Pastötter, Miriam Engel, Christian Frings

**Affiliations:** Department of Psychology, University of Trier, Trier, Germany

**Keywords:** long-term memory, episodic memory, retrieval practice, testing, learning, encoding

## Abstract

The forward effect of testing refers to the finding that retrieval practice of previously studied information increases retention of subsequently studied other information. It has recently been hypothesized that the forward effect (partly) reflects the result of a reset-of-encoding (ROE) process. The proposal is that encoding efficacy decreases with an increase in study material, but testing of previously studied information resets the encoding process and makes the encoding of the subsequently studied information as effective as the encoding of the previously studied information. The goal of the present study was to verify the ROE hypothesis on an item level basis. An experiment is reported that examined the effects of testing in comparison to restudy on items’ serial position curves. Participants studied three lists of items in each condition. In the testing condition, participants were tested immediately on non-target lists 1 and 2, whereas in the restudy condition, they restudied lists 1 and 2. In both conditions, participants were tested immediately on target list 3. Influences of condition and items’ serial learning position on list 3 recall were analyzed. The results showed the forward effect of testing and furthermore that this effect varies with items’ serial list position. Early target list items at list primacy positions showed a larger enhancement effect than middle and late target list items at non-primacy positions. The results are consistent with the ROE hypothesis on an item level basis. The generalizability of the ROE hypothesis across different experimental tasks, like the list-method directed-forgetting task, is discussed.

## Introduction

Retrieval practice of previously studied information can increase retention of subsequently studied other information, a phenomenon that has been referred to as forward effect of testing (Pastötter and Bäuml, 2014). The forward effect can be studied in a multi-list paradigm (e.g., Szpunar et al., 2008; Yang et al., 2017). In each condition, participants study several (e.g., three) lists of items. In the testing condition, participants are tested on non-target lists L1 and L2 immediately after study, whereas in the restudy condition, participants restudy L1 and L2. In both conditions, participants study and are tested on target list 3 (L3). The typical finding is that interim testing of L1 and L2 enhances recall of L3 and reduces the number of prior list intrusions in the L3 recall test. The forward effect of testing is a robust effect that has been replicated in numerous research studies employing different item materials (see Pastötter and Bäuml, 2014).

Different theoretical accounts have been suggested to explain the forward effect of testing (see Yang et al., 2018). Two prominent accounts are the release-from-proactive-interference (PI) account and the reset-of-encoding (ROE) hypothesis. The release-from-PI account assumes that interim testing of non-target lists promotes contextual list segregation, which reduces buildup of PI and facilitates recall of the target list (Szpunar et al., 2008). This view was supported by behavioral work analyzing PI-related response latencies at test (Bäuml and Kliegl, 2013). The ROE hypothesis assumes that interim testing promotes contextual list segregation, which abolishes memory load and inattentional item encoding that would build up from the encoding of earlier lists to the encoding of later lists without recall testing between lists. This ROE makes the encoding of later lists as effective as the encoding of earlier lists (Pastötter et al., 2011). The ROE hypothesis was supported by neurocognitive work. Without interim testing, oscillatory alpha power during item encoding increases with number of encoded items, both within and across item lists, a finding that has been attributed to increases in memory load and inattentional item encoding (Sederberg et al., 2006; Pastötter et al., 2008). Critically, testing between the study of item lists disrupts such alpha power increase, a finding that has been attributed to ROE (Pastötter et al., 2011).

The ROE hypothesis is not restricted to the forward effect but has been applied to other multi-list learning tasks as well. For instance, several findings suggest that ROE may play a role in list-method directed forgetting (LMDF) (see Pastötter et al., 2017). In this task, participants study two item lists and, after study of L1, receive a cue either to forget or to continue remembering this list. After study of L2, participants recall the two lists’ items irrespective of original cuing. The typical finding is that the forget cue improves recall of L2 and reduces recall of L1. The two effects have been referred to L2 enhancement and L1 forgetting (see Sahakyan et al., 2013). In LMDF, evidence for the ROE hypothesis arose from both neurocognitive and behavioral studies. The neurocognitive work provided evidence on a list level basis, demonstrating that alpha power during item encoding increases from L1 to L2 in the remember condition, but not in the forget condition (Hanslmayr et al., 2012), a finding that can be attributed to ROE. Critically, more direct evidence for the ROE hypothesis arose from behavioral studies that examined LMDF on an item level basis. Analysis of items’ serial position curves revealed that the forget cue can have a very selective enhancement effect for the early L2 items at list primacy positions, which showed larger enhancement than middle and late L2 items at non-primacy positions (Pastötter and Bäuml, 2010; Pastötter et al., 2012). Employing 12-item lists, these studies found a larger enhancement effect for L2 items at primacy positions 1–4 than for L2 items at non-primacy positions 5–12. The selective enhancement effect for the early L2 items was attributed to ROE (see also Pastötter et al., 2016; Tempel and Frings, 2016).

With regard to the forward effect of testing, current evidence for the ROE hypothesis is restricted to evidence from a neurocognitive study that examined the effects of testing on oscillatory alpha power on a list level basis (Pastötter et al., 2011). The present study aimed at providing more direct evidence for the ROE hypothesis by examining the effects of testing on an item level basis. In each condition, participants studied three 12-item lists, which they were asked to remember for final recall tests. In the testing condition, participants were tested immediately on non-target lists L1 and L2, whereas in the restudy condition, they restudied L1 and L2. In both conditions, participants were tested immediately on target L3. Based on the previous serial position findings in LMDF work and the assumption that the ROE generalizes from the enhancement effect in LMDF to the forward effect of testing, two expectations arose. First, in the testing condition, similar serial position curves and similar list primacy effects for the three item lists in the three immediate recall tests were expected. Second, in the testing compared to the restudy condition, larger enhancement for the early L3 items at list primacy positions 1–4 than for middle and late L3 items at non-primacy positions 5–12 was expected.

## Method

### Participants

Two hundred and forty students from the University of Trier participated in the study (mean age: 22.0 years, *SD* = 3.4 years; 187 females). This study was carried out in accordance with the recommendations of the local ethical review committee at the University of Trier. The protocol was approved by the committee. All participants gave written informed consent in accordance with the Declaration of Helsinki.

### Material

Item material was taken from Pastötter et al. (2012; Experiment 2), in which 144 unrelated German nouns of medium frequency and word length of 4 to 8 letters were drawn from CELEX database (Duyck et al., 2004). Nouns were assigned to six 12-item lists. The assignment of items to lists and conditions was random for each participant.

### Analyses

Proportion of correct recall was examined as a function of the within-participants factors of list (L1 to L3), serial position (primacy items 1–4, non-primacy items 5–12), and condition (testing, restudy). Items were counted as correctly recalled if recalled with the correct list. In the testing condition, L1 and L2 were tested after initial study; in the restudy condition, L1 and L2 were restudied. Plotted serial position curves were smoothed by averaging recall data over adjacent item positions (see Roediger and McDermott, 1995; Pastötter and Bäuml, 2010). The data can be downloaded at PsyArXiv^1^.

### Procedure

**FIGURE 1:**
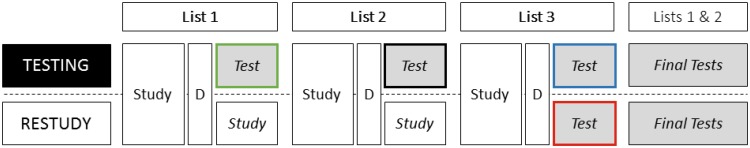
Procedure. In both the testing and the restudy condition, participants studied three lists of items. Each list consisted of 12 words and was followed by a short distractor (D). List 3 was tested immediately (after the distractor) in both conditions. Lists 1 and 2 were also tested immediately in the testing condition, but were restudied in the restudy condition. After immediate recall of list 3, lists 1 and 2 were tested in final recall tests.

Participants took part in both the testing and the restudy condition. Order of conditions was counterbalanced across participants. In both conditions, participants studied three item lists, each consisting of 12 words (see **Figure 1**). Items were visually presented in random order in the middle of a screen with a presentation rate of 3.75 s (3 s item presentation, 0.75 s blank screen). Study of each list was followed by a 30 s distractor task in which participants counted backward aloud from a three-digit number in steps of threes. In the restudy condition, L1 and L2 items were restudied with the same item presentation rate in new random order. In the testing condition, participants wrote down the items of L1 and L2 on different sheets of paper. Next, in both conditions, participants wrote down the L3 items on a new sheet of paper in the immediate L3 recall test. After that, L1 and L2 were tested in final recall tests. Recall time in each recall test was 45 s. Participants were asked to recall the words of each list in any order they wished. Following the memory experiment, working memory tasks were administered (Foster et al., 2015). The results of these working memory tasks and the relation of participants’ working memory capacity to the forward effect in the immediate L3 recall (and the effects of testing on final L1 and 2 recall) will be reported elsewhere.

## Results

### Serial Position Curves for Lists 1–3 in the Testing Condition

**FIGURE 2:**
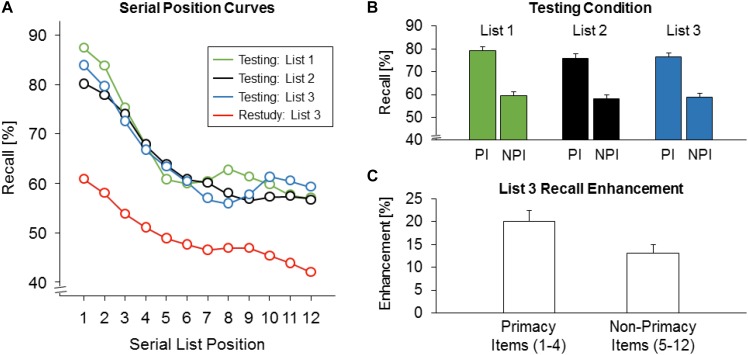
Recall results. **(A)** Serial position curves for lists 1 to 3 recall in the testing condition and for list 3 recall in the restudy condition. **(B)** Recall rates in the testing condition as a function of list (lists 1 to 3) and items’ serial list position (PI: primacy items 1 to 4, NPI: non-primacy items 5 to 12). Error bars: standard errors of the mean. **(C)** List 3 recall enhancement (testing minus restudy) as a function of items’ serial list position (primacy items 1 to 4, non-primacy items 5 to 12). Error bars: standard errors of the mean.

Serial position curves are shown in **Figure 2A**. We examined whether in the testing condition similar serial position curves and similar list primacy effects for lists 1 to 3 emerged. A 3 × 2 repeated-measures analysis of variance (r-ANOVA) with the factors of list (L1 vs. L2 vs. L3) and serial position (primacy items vs. non-primacy items) revealed a significant main effect of serial position, *F*(1,239) = 224.700, *p* < 0.001, $\eta_{p}^{2}$ = 0.485, but no significant main effect of list, *F*(2,478) = 1.736, *p* = 0.177, η$\eta_{p}^{2}$ = 0.007, nor a significant interaction between the two factors, *F*(2,478) = 0.444, *p* = 0.633, $\eta_{p}^{2}$= 0.002 (Greenhouse–Geisser corrected; **Figure 2B**). Thus, similar serial position curves and similar primacy effects for lists 1 to 3 were observed in the testing condition.

### List 3 Enhancement as a Function of Items’ Serial List Position

Next, we examined whether in the testing compared to the restudy condition a larger enhancement effect for the L3 primacy items than for the L3 non-primacy items arose. A 2 × 2 r-ANOVA with the factors of serial position (primacy items vs. non-primacy items) and condition (testing vs. restudy) revealed a significant main effect of serial position, *F*(1,239) = 104.575, *p* < 0.001, $\eta_{p}^{2}$ = 0.304, a significant main effect of condition, *F*(1,239) = 115.528, *p <* 0.001, $\eta_{p}^{2}$= 0.326, and a significant interaction between the two factors, *F*(1,239) = 5.773, *p* = 0.017, $\eta_{p}^{2}$ = 0.024. Indeed, L3 primacy items showed a larger enhancement effect (76.4 vs. 56.3%) than non-primacy items at middle and late L3 positions (59.0 vs. 45.8%; **Figure 2C**). The enhancement was reliable for both primacy and non-primacy items, *p* < 0.001, Holm-corrected.

## Discussion

The results demonstrate a reliable forward effect of testing that varied with items’ serial list position. Early L3 items at list primacy positions showed a larger enhancement effect than middle and late L3 items at non-primacy positions. In addition, in the testing condition, all three lists showed similar list primacy effects and similar serial position curves in the three immediate recall tests. Together, these results are consistent with the ROE hypothesis, which claims that testing between the study of item lists makes the encoding of later lists as effective as the encoding of earlier lists. Previous research supported the ROE hypothesis on a list level basis (Pastötter et al., 2011). Going beyond this work, the present study provides more direct evidence for the ROE hypothesis on an item level basis, indicating that ROE primarily affects the encoding and retention of early target list items at list primacy positions.

The present results suggest a parallel between the forward effect of testing and the enhancement effect in LMDF. Both interim testing and the forget instruction induce a selective enhancement effect for the early target list items, suggesting generalization of the ROE hypothesis over the two different paradigms. Importantly, in LMDF research, other factors than ROE have been suggested to contribute to the enhancement effect as well. For instance, Pastötter et al. (2012) proposed that both ROE and PI reduction can contribute to L2 enhancement. According to their two-mechanism account, ROE is restricted to early L2 items (and is present regardless of list recall order at test), and PI reduction pertains to all L2 items (and is present only if L2 is recalled first; for related findings, see Pastötter et al., 2012). Indeed, both behavioral and computational modeling work suggests that PI reduction should affect all items of the target list about equally (Davelaar et al., 2005; Neath and Brown, 2006). This provides a second parallel between the forward effect of testing and the enhancement effect in LMDF. In fact, the present results show a reliable enhancement effect for the middle and late target list items, which was smaller in size than the enhancement effect for the early list items. Following the proposal by Pastötter et al. (2012), the enhancement of the middle and late list items may reflect PI reduction. However, alternative explanations seem plausible as well. For instance, testing may change participants’ encoding strategy for subsequently studied information (Chan et al., 2018). Indeed, such change in encoding strategy should affect the encoding and retention of all target list items (regardless of list recall order at test). Therefore, it is a high priority for future work to discover exactly the interplay of (encoding and retrieval) factors that promote the forward effect of testing.

## Author Contributions

BP developed the study concept, experimental design, and drafted the manuscript. ME collected the data. BP and ME performed the data analysis. CF provided critical revisions. All authors approved the final version of the manuscript for submission.

## Conflict of Interest Statement

The authors declare that the research was conducted in the absence of any commercial or financial relationships that could be construed as a potential conflict of interest.

## Footnotes

1 https://osf.io/extrz/
